# Impact of extraction techniques on phytochemical composition and bioactivity of natural product mixtures

**DOI:** 10.3389/fphar.2025.1615338

**Published:** 2025-07-30

**Authors:** Shicai Sun, Ying Yu, Yunju Jo, Jung Ho Han, Yingqi Xue, Minkyoung Cho, Sung-Jin Bae, Dongryeol Ryu, Wonyoung Park, Ki-Tae Ha, Shiwei Zhuang

**Affiliations:** ^1^ Department of Surgery, Changchun University of Chinese Medicine, Changchun, China; ^2^ Department of Biomedical Science and Engineering, Gwangju Institute of Science and Technology, Gwangju, Republic of Korea; ^3^ Korean Medicine Application Center, Korea Institute of Oriental Medicine, Daegu, Republic of Korea; ^4^ Department of Parasitology and Tropical Medicine, and Institute of Health Sciences, Gyeongsang National University College of Medicine, Jinju, Republic of Korea; ^5^ Department of Molecular Biology and Immunology, Kosin University College of Medicine, Busan, Republic of Korea; ^6^ Department of Korean Medical Science, School of Korean Medicine, Pusan National University, Yangsan, Republic of Korea; ^7^ Research Institute for Korean Medicine, Pusan National University, Yangsan, Republic of Korea

**Keywords:** extraction techniques, natural products, bioactive compounds, phytochemical composition, pharmaceutical and nutraceutical applications

## Abstract

Extraction methods critically influence the phytochemical profile and bioactivity of natural product mixtures, affecting their efficacy as therapeutic agents. This review assesses the impact of various extraction techniques—such as solvent-based extraction, ultrasound-assisted extraction, and enzymatic extraction—on the composition of bioactive compounds in plant extracts. We discuss how extraction parameters modify the bioactivity profiles, influencing their application in pharmaceutics and nutrition. This review critically analyzes these techniques with a special focus on integrated (hybrid) strategies, revealing that while advanced methods like ultrasound-assisted extraction and microwave-assisted extraction offer improved efficiency, the greatest potential lies in the synergistic combination of methods to maximize yield and preserve bioactivity. The insights gathered here aim to guide researchers in developing optimal and sustainable extraction protocols for therapeutic applications.

## 1 Introduction

Natural products, particularly plant-derived extracts, have garnered immense interest due to their wide range of bioactivities and potential therapeutic applications (Tomlinson and Akerele, 2015). They have been utilized in traditional medicine for centuries, and modern research continues to highlight their roles in treating various diseases, including inflammatory disorders, metabolic syndromes, and even cancer (Gurib-Fakim, 2006). The bioactive compounds within these extracts—such as polyphenols, flavonoids, alkaloids, terpenoids, and glycosides—exhibit diverse pharmacological activities, including antioxidant, anti-inflammatory, antimicrobial, and anticancer properties (Shanmugam et al., 2021). Consequently, these natural bioactives are increasingly incorporated into pharmaceuticals, nutraceuticals, functional foods, and even cosmetic formulations (da Silva et al., 2016a). However, to fully harness their therapeutic potential, an optimized extraction process is essential to ensure the stability, yield, and bioactivity of these compounds (Bhadange et al., 2024).

The choice of extraction method plays a crucial role in determining the chemical composition and bioactivity of natural product mixtures (Doughari, 2012). Traditional extraction methods, such as maceration, Soxhlet extraction, and hydrodistillation, have long been used to isolate bioactive compounds from plant materials (Yolci Omeroglu et al., 2019). While these methods are relatively simple and cost-effective, they often suffer from major drawbacks, including low efficiency, long extraction times, high solvent consumption, and potential degradation of heat-sensitive compounds like flavonoids and polyphenols (Luksta and Spalvins, 2023). These limitations have driven the development of advanced extraction techniques, including ultrasound-assisted extraction (UAE), microwave-assisted extraction (MAE), supercritical fluid extraction (SFE), and enzyme-assisted extraction (EAE) (Ahmad et al., 2025). These modern techniques offer significant advantages such as higher extraction yields, improved selectivity, reduced processing time, and better preservation of bioactive integrity (Usman et al., 2023).

Extraction efficiency is influenced by multiple parameters, including solvent type, pH, temperature, extraction duration, particle size, and mechanical forces applied (Spietelun et al., 2013). For example, polar solvents (e.g., methanol, ethanol, and water) are commonly used to extract hydrophilic compounds like phenolics and flavonoids, whereas non-polar solvents (e.g., hexane and chloroform) are more effective for lipophilic compounds such as terpenoids and carotenoids (Sicker et al., 2019). Additionally, innovative approaches like EAE allow for the selective breakdown of plant cell walls, facilitating the release of intracellular bioactive compounds while minimizing degradation (Sousa et al., 2023). Understanding how these parameters influence phytochemical composition is critical for optimizing extraction protocols (Doughari, 2012).

A major challenge in the field of natural product extraction is standardization (Kunle et al., 2012). The phytochemical composition of extracts can vary significantly depending on plant species, geographic origin, environmental conditions, and harvesting time, making it difficult to ensure batch-to-batch consistency (Atanasov et al., 2021). This variability poses a significant issue for pharmaceutical and nutraceutical applications, where bioactivity and safety must be precisely controlled (Butler, 2004). To address these challenges, advanced analytical techniques such as high-performance liquid chromatography (HPLC), gas chromatography-mass spectrometry (GC-MS), and nuclear magnetic resonance (NMR) spectroscopy have been employed to provide detailed chemical profiling and quality assessment of natural extracts (SL Mendez et al., 2015). These analytical tools not only improve reproducibility but also help in identifying the most effective extraction methods for preserving bioactive compounds (Altemimi et al., 2017).

Given the increasing demand for bioactive natural products in various industries, there is a critical need for systematic evaluations of extraction techniques (Patra et al., 2018). This review aims to comprehensively assess the impact of different extraction methods on the yield, phytochemical composition, and bioactivity of plant-based extracts. By comparing conventional and advanced extraction techniques, we highlight the advantages, limitations, and potential applications of each method. Additionally, we explore the role of analytical technologies in ensuring standardized and high-quality natural product extracts. Through this discussion, we aim to contribute valuable insights toward the development of sustainable and efficient extraction strategies that enhance the therapeutic and commercial applications of bioactive natural compounds. While numerous reviews have summarized individual extraction techniques, this paper provides a unique contribution by critically analyzing the synergies and trade-offs of integrated (hybrid) extraction strategies.

### 1.1 Literature search strategy and scope of the review

This article provides a critical narrative review of the literature concerning extraction techniques for natural products. It is not intended to be a systematic review following PRISMA guidelines, but rather a comprehensive overview to identify current trends, challenges, and future perspectives. The literature was surveyed using major scientific databases, including Scopus, PubMed, and Google Scholar, with a primary focus on publications from the last 2 decades to cover recent advancements. However, seminal, highly-cited foundational papers from before this period were also included to provide essential historical context. Keywords used for the search included, but were not limited to: “natural product extraction,” “phytochemicals,” “bioactivity,” “ultrasound-assisted extraction,” “microwave-assisted extraction,” “supercritical fluid extraction,” “hybrid methods,” and “synergy.” The primary inclusion criterion was peer-reviewed research and review articles published in English that provided significant insights into extraction principles, applications, and challenges. Conference abstracts, patents, and non-English articles were excluded from this review.

## 2 Impact of extraction techniques on phytochemical composition and bioactivity

The efficacy of bioactive compounds derived from natural products is highly dependent on the extraction technique employed (Wijngaard et al., 2012). The choice of method influences the yield, stability, and pharmacological activity of phytochemicals, affecting their antioxidant, anti-inflammatory, antimicrobial, and therapeutic potential (Tauro et al., 2024). Various chemical, physical, and enzymatic factors contribute to extraction efficiency, including solvent type, temperature, pH, extraction duration, and mechanical forces (Wen et al., 2020). These factors not only dictate the quantity of bioactive compounds recovered but also their structural integrity and bioactivity (Ventura et al., 2017).

### 2.1 Influence of extraction techniques on phytochemical composition

Extraction methods significantly impact the solubility, stability, and concentration of bioactive compounds (Zhang et al., 2020). Conventional approaches, which are discussed in detail in Section 3, include traditional methods such as maceration, Soxhlet extraction, and hydrodistillation. While these techniques have been used for decades, they often suffer from low efficiency, high solvent consumption, and long extraction times (Dhobi et al., 2009). Moreover, heat-sensitive compounds such as polyphenols, flavonoids, and terpenoids are prone to degradation under prolonged exposure to high temperatures (Valisakkagari et al., 2024).

Modern advanced extraction techniques have been developed to overcome these limitations by enhancing extraction efficiency, reducing solvent use, and preserving bioactive integrity (Khaw et al., 2017). Depending on the method used, the composition of extracted phytochemicals can vary significantly (Kumar et al., 2023):

Solvent-based extractions are highly dependent on polarity, with polar solvents (e.g., ethanol, water) favoring hydrophilic compounds (e.g., flavonoids, tannins) and non-polar solvents (e.g., hexane, chloroform) extracting lipophilic bioactives (e.g., terpenoids, carotenoids) (Da Silva et al., 2022).

Mechanically-assisted extractions (e.g., ultrasound and microwave methods) enhance cell wall disruption, facilitating the release of intracellular compounds while minimizing structural degradation (Rocchetti et al., 2022).

Enzyme-assisted methods improve the selective extraction of glycosides, polysaccharides, and other cell wall-associated compounds, increasing bioavailability (Wang et al., 2024).

### 2.2 Effect of extraction techniques on bioactivity

The biological activity of plant extracts is influenced not only by the presence of bioactive compounds but also by their structural stability and bioavailability, which extraction methods affect (Usman et al., 2022). Studies comparing different extraction techniques have shown that optimized methods lead to higher antioxidant, anti-inflammatory, and antimicrobial effects due to the enhanced recovery of functional phytochemicals (Kumar et al., 2023).

The superiority of modern techniques is well-illustrated by flavonoid extraction from citrus peels. Conventional Soxhlet extraction requires prolonged heating at the solvent’s boiling point (e.g., ∼78°C for ethanol), which can cause thermal degradation of sensitive compounds (Ćujić et al., 2016). In contrast, UAE utilizes acoustic cavitation at lower temperatures, enabling a more efficient recovery of these phytochemicals (Chemat et al., 2017). Consequently, UAE extracts from citrus peels consistently show higher yields of flavonoids and superior antioxidant activity (Khan et al., 2010). This is therapeutically significant, as these flavonoids, such as hesperidin, are known to possess potent anti-inflammatory effects which are compromised by heat (Parhiz et al., 2015; Al-Khayri et al., 2022).

Antioxidant Properties: Extraction techniques that efficiently retain polyphenols and flavonoids result in higher free radical scavenging potential, reducing oxidative stress (Brglez Mojzer et al., 2016).

Anti-Inflammatory Effects: Enhanced recovery of terpenoids and phenolic acids con-tributes to modulation of pro-inflammatory pathways [e.g., inhibition of Nuclear Factor-kappa B (NF-κB), Cyclooxygenase-2 (COX-2) enzymes] (da Cunha et al., 2022).

Antimicrobial Properties: The preservation of alkaloids, tannins, and saponins through optimized extraction enhances their antibacterial and antifungal effects, preventing microbial contamination in food and pharmaceutical applications (Oulahal and Degraeve, 2022).

Additionally, extraction efficiency is linked to particle size, solvent polarity, and processing conditions. For example:

Reducing particle size increases the surface area for solvent penetration, improving yield (Singh, 2017).

Selecting the appropriate solvent ensures maximum solubility of the target compounds while minimizing unwanted co-extractions (Reis et al., 2020).

### 2.3 Challenges in standardizing extraction techniques

Although advanced extraction methods improve efficiency and bioactivity, achieving batch-to-batch consistency remains a challenge. Several factors contribute to variations in extract quality:

Raw Material Variability: Differences in geographical origin, cultivation practices, and harvesting conditions affect phytochemical composition (Tiwari and Cummins, 2013).

Processing Inconsistencies: Variability in temperature, solvent selection, and extraction duration influences final extract quality (Rajha et al., 2014).

Regulatory Compliance: Standardization is crucial for pharmaceutical and nutraceutical applications, necessitating the use of analytical validation techniques (HPLC, GC-MS, NMR) (Durazzo et al., 2022).

### 2.4 Integrated (hybrid) extraction strategies: synergies and trade-offs

Emerging research suggests that combining multiple extraction techniques can maximize compound yield and functional properties. The rationale behind integrated or “hybrid” extraction strategies is to leverage the unique advantages of multiple techniques to create a synergistic effect that a single method cannot achieve (Chemat et al., 2019a). A prime example is the sequential combination of EAE and UAE. EAE can be initially employed to enzymatically degrade the rigid cellulose and pectin matrix of the plant cell wall, creating a more porous structure (Muniglia et al., 2014). Subsequently, the acoustic cavitation generated by UAE can penetrate this pre-weakened matrix more effectively, leading to a dramatic increase in the release of intracellular bioactive compounds (Kumar et al., 2021). This synergy allows for higher yields at lower temperatures and shorter times than either EAE or UAE could accomplish alone (Puri et al., 2012; Xiong et al., 2019).

In addition to this EAE-UAE combination, other hybrid approaches have also demonstrated significant potential. These include:

Microwave-assisted hydrodistillation (MAHD): This method integrates microwave heating into the hydrodistillation process. The rapid, internal heating generated by microwaves shortens the distillation time significantly and can improve the yield and quality of essential oils compared to traditional hydrodistillation alone (Lucchesi et al., 2004; Golmakani and Rezaei, 2008).

Supercritical fluid extraction with a co-solvent (SFE-CO_2_ w/co-solvent): While not a sequential hybrid method, this approach “integrates” a polar co-solvent (like ethanol) into the non-polar SC-CO_2_ fluid. This dramatically enhances the extraction efficiency for moderately polar compounds, such as certain flavonoids and phenolic acids, which are poorly soluble in pure SC-CO_2_ (Gallego et al., 2019; Paucar et al., 2023).

However, these integrated approaches are not without significant trade-offs that require critical consideration. Combining multiple techniques inevitably increases process complexity, the number of parameters to optimize, and overall operational costs, which can be a barrier for industrial scale-up (Nde and Foncha, 2020; Ahangari et al., 2021). For instance, a hybrid SFE-ultrasound system requires high-pressure equipment coupled with a high-power ultrasonic transducer, representing a substantial capital investment (De Melo et al., 2014). Furthermore, a multi-step process introduces additional potential points of failure and may increase total processing time, even if individual steps are rapid. Therefore, the decision to implement a hybrid system must be justified by a significant and measurable improvement in yield, purity, or bioactivity that outweighs these practical and economic challenges (Chemat et al., 2019a).

## 3 Conventional extraction methods and their limitations

Traditional extraction techniques, such as maceration, Soxhlet extraction, and hydrodistillation, have long been used to obtain bioactive compounds from plant materials (Yolci Omeroglu et al., 2019). These methods rely on physical and chemical interactions between the solvent and plant matrix to extract target compounds (Ingle et al., 2017). While conventional techniques are widely utilized due to their simplicity and cost-effectiveness, they also present significant limitations, including long extraction times, high solvent consumption, thermal degradation, and lack of selectivity (Manousi et al., 2019).

### 3.1 Maceration

Maceration is one of the simplest and most commonly used extraction methods, involving soaking plant material in a suitable solvent at room temperature for an extended period (Hidayat and Wulandari, 2021). This technique allows for the passive diffusion of bioactive compounds into the solvent (Chongo, 2025). However, it is highly time-consuming and often leads to incomplete extraction (Shikov et al., 2022). Additionally, maceration may result in the co-extraction of undesirable compounds, affecting the purity and bioactivity of the final extract (Chuo et al., 2022).

### 3.2 Soxhlet extraction

Soxhlet extraction is a continuous solvent extraction technique that involves repeated percolation of hot solvent over the plant material (López-Bascón and De Castro, 2020). This method increases extraction efficiency compared to maceration by continuously replenishing the solvent, allowing for better solubility and diffusion of target compounds (Şahin et al., 2011). However, its major drawback is the prolonged exposure to high temperatures, which can degrade heat-sensitive bioactive compounds such as polyphenols and flavonoids (Antony and Farid, 2022). Additionally, Soxhlet extraction requires a large volume of solvent, making it less environmentally friendly and cost-effective (Trolles-Cavalcante et al., 2021).

### 3.3 Hydrodistillation

**FIGURE 1:**
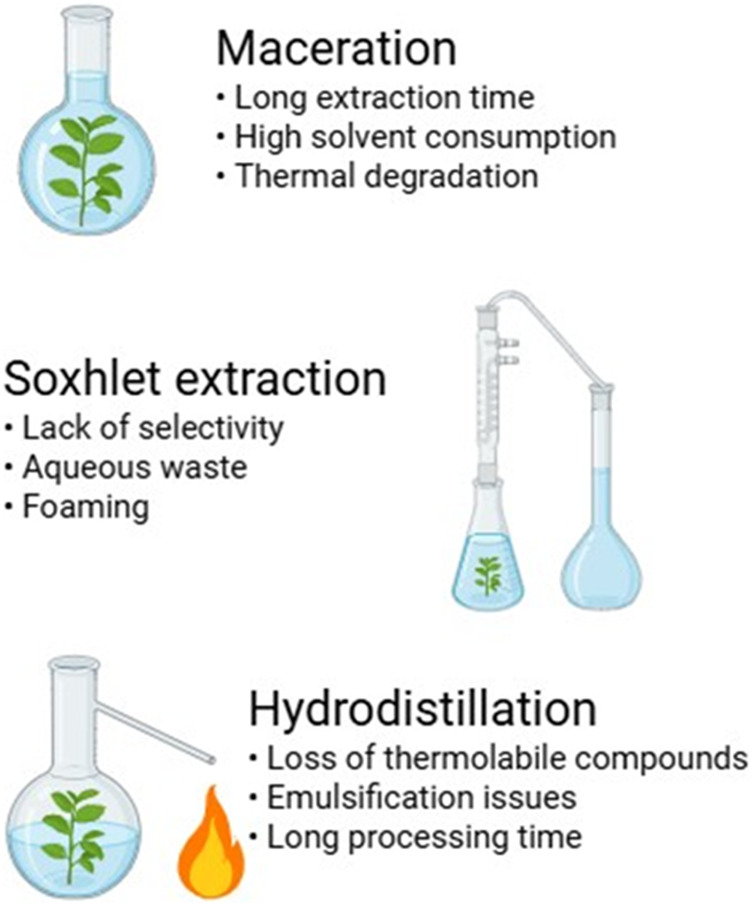
Limitations of traditional extraction techniques.

Hydrodistillation is primarily used for extracting essential oils and volatile compounds from plant materials (Abbas et al., 2017). This method involves boiling plant material in water or steam to release volatile compounds, which are then condensed and collected (Perović et al., 2024). While effective for obtaining essential oils, hydrodistillation poses challenges such as the loss of thermolabile compounds, emulsification issues, and long processing times (Pheko-Ofitlhile and Makhzoum, 2024). Furthermore, the high temperatures used in hydrodistillation can alter the chemical composition of extracted oils, affecting their bioactivity and fragrance profile (Lainez-Cerón et al., 2021) (Figure 1).

## 4 Factors influencing extraction efficiency and bioactivity

The efficiency of bioactive compound extraction and the biological activity of the final extract depend on multiple physicochemical and process-related factors (Da Silva R. P. et al., 2016). These factors influence the yield, stability, solubility, and pharmacological effectiveness of extracted phytochemicals. Optimizing these parameters is essential to ensure that bioactive compounds are recovered efficiently while maintaining their structural integrity.

### 4.1 Solvent selection

Solvent choice is one of the most critical factors in determining extraction efficiency. The polarity of the solvent plays a key role in selecting specific classes of bioactive compounds:

Polar solvents (e.g., water, ethanol, methanol) efficiently extract hydrophilic compounds such as flavonoids, polyphenols, and anthocyanins (Tzanova et al., 2020).

Non-polar solvents (e.g., hexane, chloroform, ethyl acetate) are better suited for lipophilic bioactives such as terpenoids, carotenoids, and essential oils (Siddiqui et al., 2024).

Binary or ternary solvent systems (e.g., ethanol-water mixtures) enhance the extraction of a broad range of phytochemicals, improving both yield and bioactivity (Natolino et al., 2024).

The critical role of solvent polarity and the benefits of mixed-solvent systems are well-illustrated by the extraction of polyphenols from green tea (*Camellia sinensis*). For instance, a study demonstrated that using a 70% ethanol-water mixture yielded significantly higher total phenolic content and antioxidant activity compared to using either pure water or absolute ethanol (Calderón-Oliver and Ponce-Alquicira, 2021; Maslov et al., 2022). Pure water primarily extracts highly polar compounds, while absolute ethanol is less effective for these (Cheng et al., 2021). The ethanol-water mixture, however, creates a solvent environment with intermediate polarity, capable of efficiently extracting a wider spectrum of polyphenols, which in turn leads to enhanced synergistic bioactivity (Plaskova and Mlcek, 2023). This highlights the necessity of optimizing solvent systems to match the polarity of the target bioactive compounds.

In recent years, green solvents (defined as environmentally benign solvents, typically derived from renewable resources, that are characterized by low toxicity, biodegradability, and high efficiency) have gained attention for their eco-friendly and efficient extraction capabilities. Among these, Deep Eutectic Solvents (DES), typically formed from natural compounds like choline chloride and organic acids, are emerging as promising alternatives to volatile organic solvents (Dai et al., 2013; Prabhune and Dey, 2023; Stanisz et al., 2024; Suthar, 2025). For example, a study on the extraction of anthocyanins from grape skin showed that a specific DES formulation achieved a higher extraction yield and resulted in an extract with greater antioxidant stability compared to conventional acidified ethanol, all while being biodegradable and non-toxic (Jeong et al., 2015; Bosiljkov et al., 2017; Foroutani et al., 2024). This demonstrates the potential of green solvents to not only reduce environmental impact but also, in some cases, enhance the quality and stability of the extracted bioactive compounds (Chemat et al., 2019b; Aduloju et al., 2023; Ferreira and Sarraguça, 2024).

### 4.2 Temperature and extraction time

Both temperature and extraction time significantly affect the yield and stability of bioactive compounds:

Higher temperatures can increase solubility, diffusion rates, and mass transfer, leading to improved extraction efficiency (Kuosmanen et al., 2003).

However, prolonged exposure to high temperatures may cause oxidative degradation of thermolabile compounds such as vitamin C, polyphenols, and essential oils (Awad et al., 2021).

For instance:

Flavonoids and phenolic acids exhibit enhanced extraction at moderate temperatures (40°C–60°C), but temperatures above 80°C may lead to degradation (Antony and Farid, 2022).

Terpenoids and alkaloids, which are more stable, can be extracted efficiently at higher temperatures (70°C–100°C) (Rasul, 2018).

Supercritical CO_2_ extraction operates at relatively low temperatures, making it ideal for preserving heat-sensitive compounds while maintaining high efficiency (Wang et al., 2021).

The extraction duration also plays a crucial role in maximizing bioactive compound recovery (Belščak-Cvitanović and Komes, 2017). Extended extraction times can increase yield but may lead to oxidation, hydrolysis, or polymerization, altering the bioactivity of sensitive phytochemicals (Brglez Mojzer et al., 2016).

### 4.3 pH and enzyme-assisted extraction

The pH of the extraction medium can influence solubility, ionization, and stability of bioactive compounds:

Acidic conditions (pH < 5) favor the extraction of anthocyanins and organic acids but may cause degradation of certain polyphenols (Friedman and Jürgens, 2000; Enaru et al., 2021).

Alkaline conditions (pH > 7) enhance the solubility of alkaloids but may degrade phenolic compounds (Yubin et al., 2014; Oreopoulou et al., 2019).

EAE enhances extraction efficiency by breaking down plant cell walls and releasing intracellular bioactives (Wijesinghe and Jeon, 2012):

Cellulases and pectinases degrade plant fiber matrices, facilitating the release of flavonoids and glycosides (Costa et al., 2020).

Proteases can help recover bioactive peptides from protein-rich plant materials (David Troncoso et al., 2022).

Hemicellulases improve the extraction of arabinoxylans and other polysaccharides (de Souza and Kawaguti, 2021).

EAE is particularly useful in the food and pharmaceutical industries, as it reduces the need for harsh solvents and improves bioavailability.

### 4.4 Particle size and pretreatment methods

Reducing the particle size of plant material can enhance solvent penetration and increase surface area, improving extraction efficiency (Ameer et al., 2017). Various pretreatment techniques have been employed to optimize extraction:

Grinding and milling: Reduces particle size, enhancing mass transfer (Kratky and Jirout, 2011).

Freeze-drying: Preserves bioactive integrity while facilitating better solvent diffusion (Rezvankhah et al., 2020).

Microwave and ultrasound pretreatment: Improves cell wall disruption, leading to higher yields (Passos et al., 2015).

### 4.5 Advanced extraction optimization strategies

To maximize extraction efficiency, various optimization approaches have been explored:

Multistage extraction: Sequentially using different solvents to target multiple bioactive groups (Wen et al., 2020).

Solvent recycling and green extraction: Employing eco-friendly solvents and low-energy extraction methods to reduce environmental impact (Hessel et al., 2022).

Automated process control: Using real-time monitoring and AI-driven optimization to improve reproducibility and efficiency (Yingngam et al., 2024).

**FIGURE 2:**
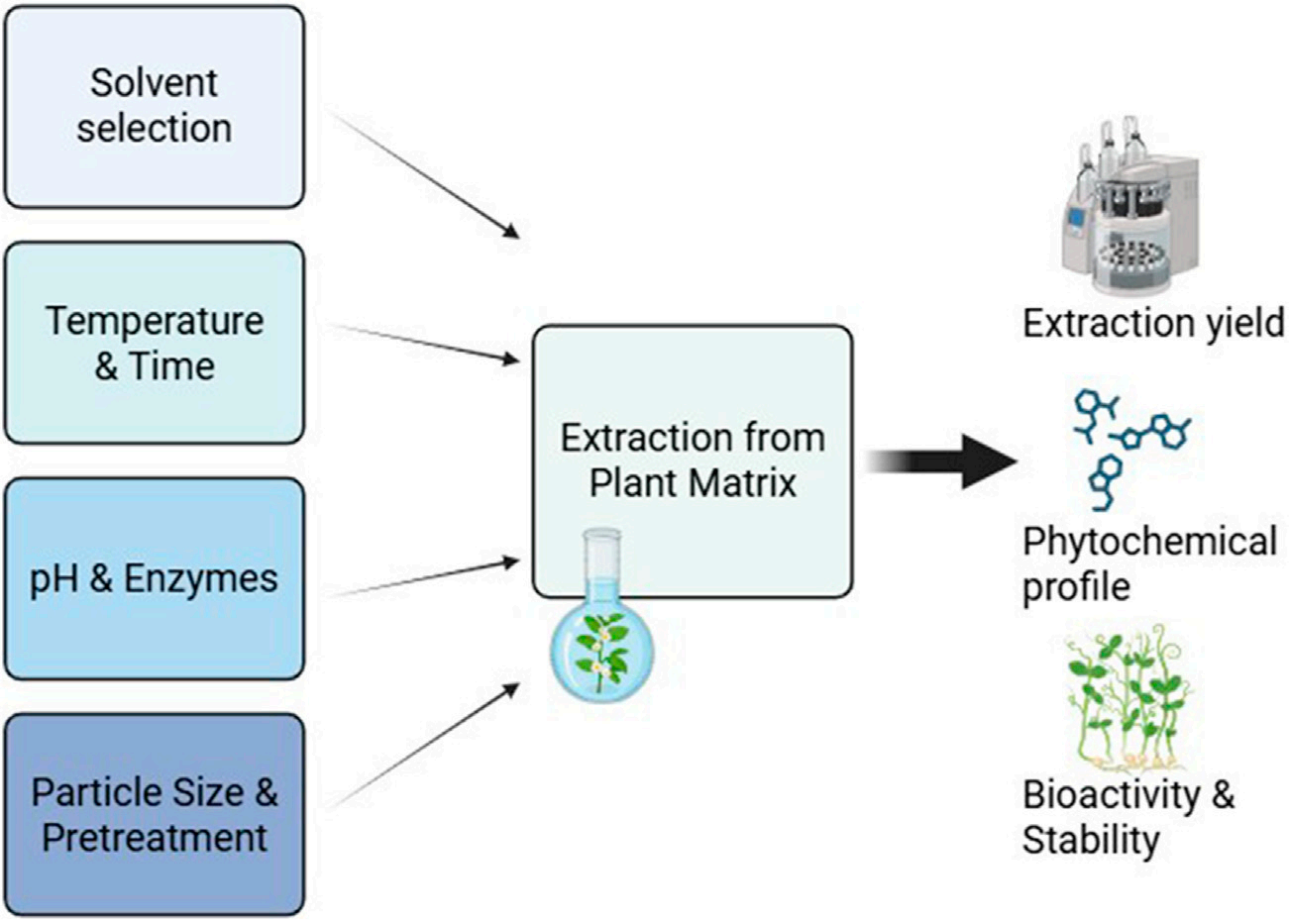
Key parameters influencing extraction efficiency and bioactivity.

By understanding and optimizing these factors, researchers can significantly improve the yield, stability, and bioactivity of natural product extractions, leading to more effective pharmaceutical, nutraceutical, and functional food applications (Figure 2).

## 5 Advanced extraction techniques for enhanced bioactivity

Advanced extraction techniques have been developed to address the limitations of conventional methods, offering improved efficiency, selectivity, and preservation of bioactive compounds (Zia et al., 2022). These techniques leverage modern technologies to enhance the recovery of bioactive molecules while minimizing degradation and solvent usage (Rifna et al., 2023).

### 5.1 Ultrasound-assisted extraction

UAE employs high-frequency sound waves to create cavitation, which disrupts plant cell walls and facilitates the release of intracellular compounds (Islam et al., 2023). This method enhances the extraction efficiency of phenolics, flavonoids, and other bioactive molecules while reducing extraction time and solvent consumption (Lovrić et al., 2017; Anaya-Esparza et al., 2023; Demesa et al., 2024).

### 5.2 Microwave-assisted extraction

MAE uses microwave radiation to rapidly heat the solvent and plant matrix, increasing the diffusion of target compounds. This technique has been shown to improve the recovery of thermolabile bioactives and enhance antioxidant potential compared to conventional extraction methods (Kanitkar et al., 2011).

### 5.3 Supercritical fluid extraction

SFE, particularly with supercritical carbon dioxide (SC-CO_2_), is effective for extracting non-polar compounds such as terpenoids and lipophilic antioxidants (Dashtian et al., 2024). The adjustable pressure and temperature conditions allow for selective extraction while reducing solvent residues in the final product (Herrero et al., 2010). These advanced techniques provide promising alternatives to conventional extraction methods, ensuring higher efficiency and improved bioactivity of natural product extracts (Khaw et al., 2017).

### 5.4 Critical considerations and parameter sensitivity

**FIGURE 3:**
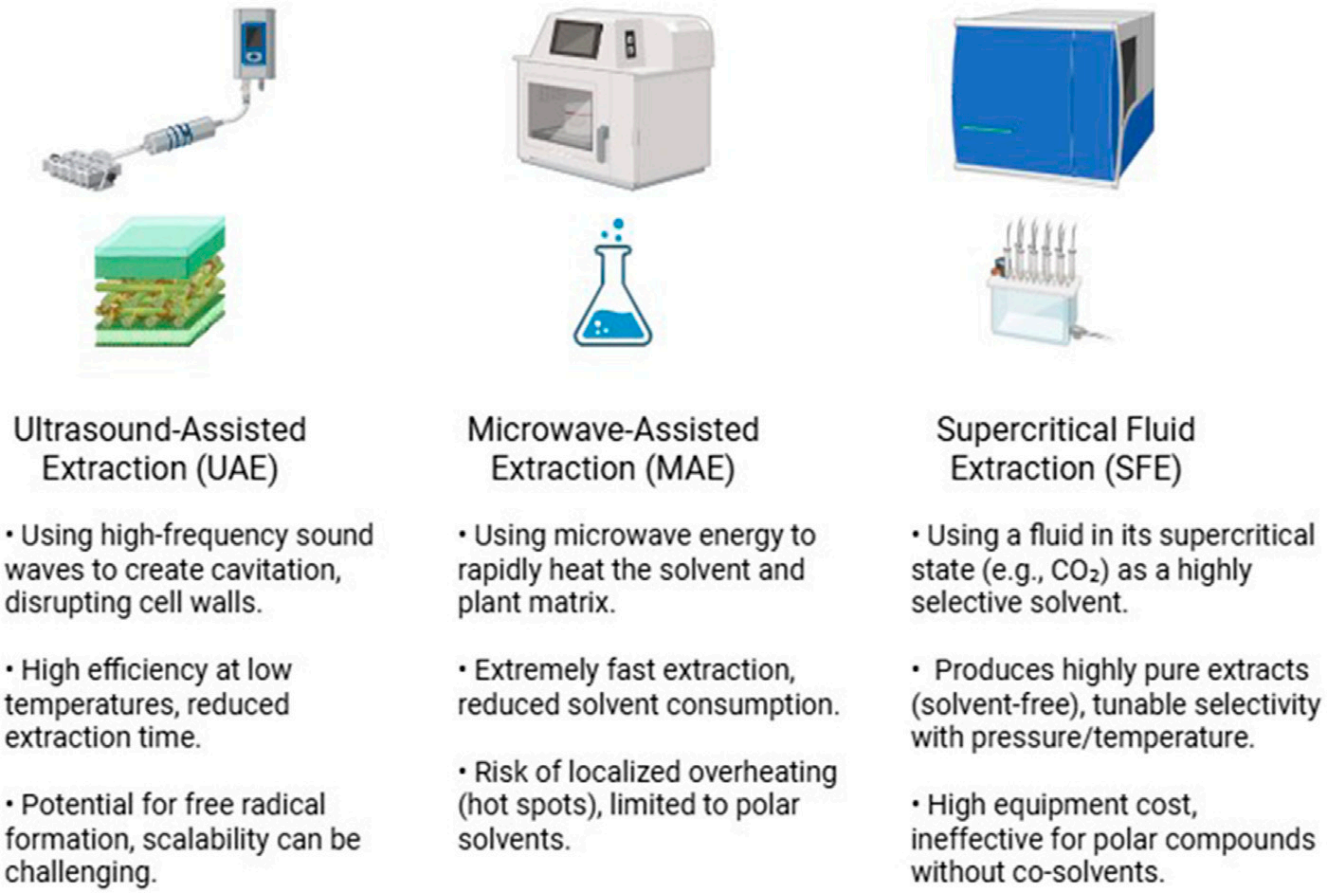
Comparison of principles and features of advanced extraction methods.

While advanced techniques like UAE and MAE are widely praised for their high efficiency and reduced processing times, it is crucial to recognize that their effectiveness is not universal and is highly parameter-dependent. A critical review of the literature reveals that direct comparisons can yield variable outcomes, highlighting that there is no single “best” method for all applications (Azwanida, 2015). This variability often stems from the sensitivity of these methods to processing parameters. For example, while moderate sonication power in UAE can enhance cell disruption, excessive power or prolonged exposure can generate free radicals, leading to the degradation of thermolabile compounds like certain flavonoids (Shirsath et al., 2012; Chemat et al., 2017). In such cases, the final extract may exhibit lower bioactivity than one obtained from a carefully optimized conventional method (Kumar et al., 2021). Similarly, the effectiveness of MAE is highly dependent on the dielectric properties of the solvent and the matrix (Camel, 2000; Vinatoru et al., 2017). Improper settings can cause uneven heating and “hot spots,” which can compromise compound integrity (Mandal et al., 2007; Chemat et al., 2020; Bhadange et al., 2024). Therefore, a more nuanced perspective is required. The selection of an advanced technique must be accompanied by rigorous optimization of its key parameters (e.g., power, temperature, time, solvent choice) tailored to the specific phytochemicals and plant matrix (Bezerra et al., 2008). Simply adopting an advanced method without this critical optimization does not guarantee a superior result (Figure 3; Table 1).

**TABLE 1:** Comparison of conventional extraction methods.

Method	Principle	Processing time	Relative yield	Energy use	Thermal stability	Selectivity	Initial cost
Maceration	Soaking material in solvent at room temperature (Othman et al., 2024)	Very Long	Low	Very Low	Good	Low	Low
Soxhlet Extraction	Continuous washing with fresh, hot, refluxing solvent (Malabadi et al., 2024)	Long	Moderate-High	High	Poor	Low-Moderate	Low
Hydrodistillation	Co-distillation of volatile compounds with boiling water or steam (Paini et al., 2025)	Long	Variable (High for volatiles)	High	Poor	High (for volatiles)	Low
UAE	Disrupts cell walls using high-frequency acoustic cavitation (Sethi and Rathod, 2024)	Short	High	Low-Medium	Good	Moderate	Medium
MAE	Uses microwave energy for rapid, direct heating of the matrix (Dhotre, 2025)	Very Short	High	Medium	Fair	Moderate-High	Medium
SFE	Uses a fluid (mainly CO_2_) in its supercritical state as a solvent (Herzyk et al., 2024)	Medium	High	High	Excellent	High	High

## 6 Analytical techniques for phytochemical profiling

The accurate identification and quantification of phytochemicals in plant extracts are essential for evaluating their bioactivity and therapeutic potential (Doughari, 2012). Various analytical techniques have been developed to profile the complex composition of natural product mixtures, allowing researchers to assess extraction efficiency, standardize bioactive compounds, and ensure batch-to-batch consistency (Nikam et al., 2012).

### 6.1 High-performance liquid chromatography

HPLC is a widely used analytical method for separating, identifying, and quantifying phytochemicals in plant extracts (Boligon and Athayde, 2014). It operates by passing a liquid mobile phase through a column packed with a stationary phase, where compounds interact based on their polarity and affinity (Žuvela et al., 2019). This technique is effective for detecting flavonoids, polyphenols, alkaloids, and glycosides (Wolfender, 2009; Yuan et al., 2019).

Application: Used extensively in pharmaceutical and nutraceutical industries for quality control and standardization of plant extracts (Choudhary and Sekhon, 2011).

Advantages: High resolution, sensitivity, and reproducibility.

Limitations: Requires extensive sample preparation and solvent use (Dong, 2013).

### 6.2 Gas chromatography-mass spectrometry

Gas Chromatography-Mass Spectrometry (GC-MS) is ideal for analyzing volatile and semi-volatile phytochemicals, such as essential oils, terpenoids, and fatty acids (Ye, 2009). It works by vaporizing a sample and passing it through a column where compounds are separated based on their volatility (Hubschmann, 2025). The separated compounds are then identified based on their mass-to-charge ratio (Xie et al., 2019).

Application: Used in essential oil profiling, fragrance analysis, and food quality testing (Song and Liu, 2018).

Advantages: High specificity, rapid analysis, and excellent compound identification (Hao et al., 2007).

Limitations: Not suitable for non-volatile or thermally unstable compounds (Kornilova et al., 2013; Beale et al., 2018).

### 6.3 Nuclear magnetic resonance spectroscopy

NMR spectroscopy provides structural and compositional insights into phytochemicals by detecting the interactions of atomic nuclei with an external magnetic field (Gautam et al., 2025). Unlike chromatographic methods, NMR is non-destructive and provides a comprehensive overview of molecular structures (Cade-Menun, 2005).

Application: Used for metabolomics studies, structural elucidation, and complex mixture analysis (Letertre et al., 2020).

Advantages: No extensive sample preparation, provides detailed molecular information.

Limitations: High cost of equipment and expertise required for data interpretation (Marcone et al., 2013).

### 6.4 Fourier Transform Infrared spectroscopy

Fourier Transform Infrared (FTIR) spectroscopy identifies functional groups in phytochemicals based on their absorption of infrared light (Vijayalakshmi and Ravindhran, 2012). It provides molecular fingerprints that are useful in distinguishing different plant metabolites (Rebiai et al., 2022).

Application: Used for rapid quality control, authentication of herbal medicines, and detecting adulteration (Huck, 2015).

Advantages: Fast, cost-effective, and non-destructive (Kumari et al., 2018).

Limitations: Limited ability to distinguish structurally similar compounds (Baker et al., 2014).

### 6.5 Ultra-high-performance liquid chromatography

Ultra-High-Performance Liquid Chromatography (UHPLC) is an advanced form of HPLC that operates under higher pressure, allowing for faster separation and improved resolution (Dong and Zhang, 2014). This technique is particularly beneficial for analyzing complex phytochemical mixtures and detecting minor bioactive constituents (Wu et al., 2013).

Application: Used in pharmaceutical analysis, high-throughput screening, and quality control of functional foods (Ahmed et al., 2023).

Advantages: Increased resolution, faster analysis time, and lower solvent consumption (Dong and Zhang, 2014).

Limitations: Higher instrument cost and maintenance requirements (Dong and Zhang, 2014).

**FIGURE 4:**
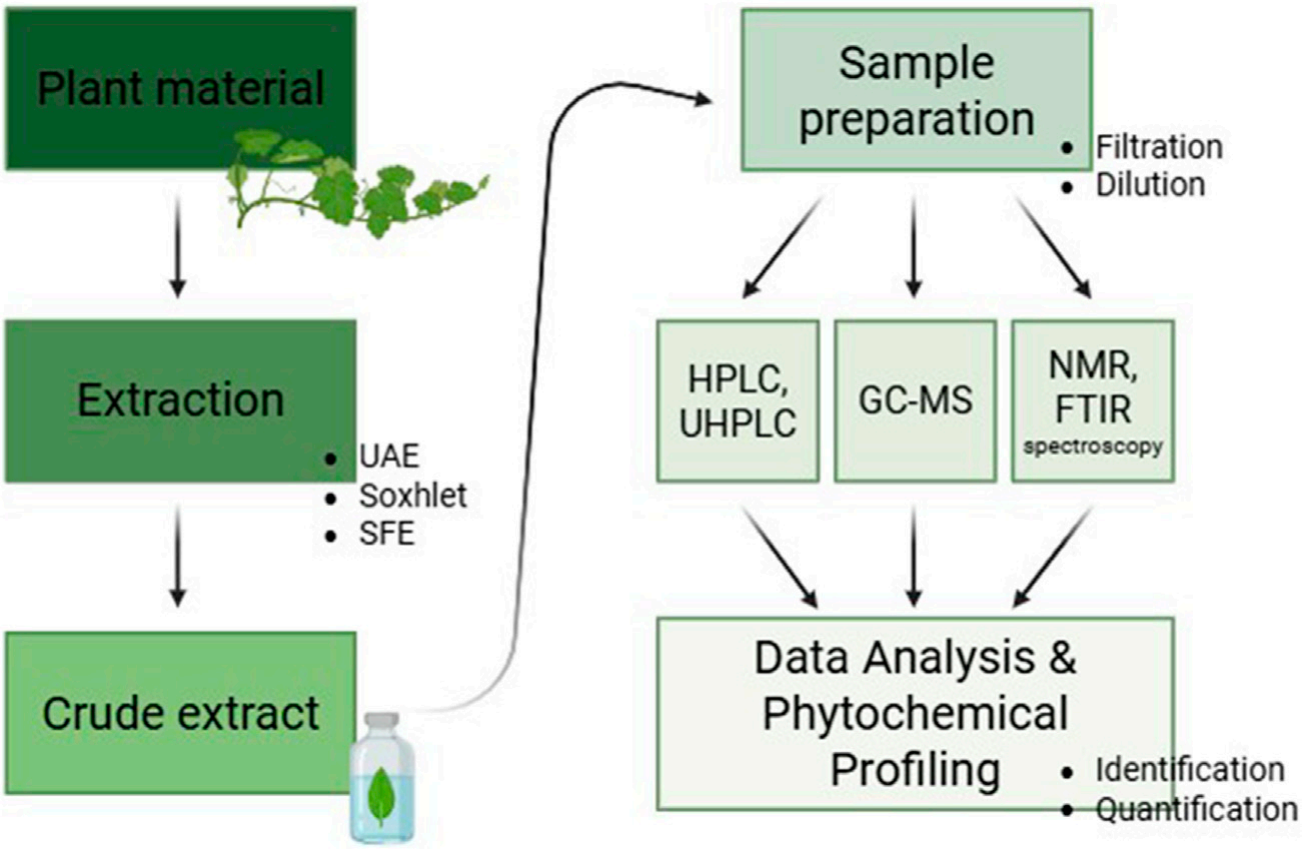
General workflow for phytochemical profiling of plant extracts.

These analytical techniques are essential for accurately profiling phytochemicals and ensuring the quality and efficacy of natural product extracts. The choice of method depends on the nature of the compounds being analyzed, as well as the intended application. By integrating multiple techniques, researchers can achieve a comprehensive understanding of phytochemical composition and bioactivity, leading to improved standardization in pharmaceuticals, nutraceuticals, and functional foods (Figure 4).

## 7 Challenges and future perspectives in standardizing and scaling up extraction methods

Despite significant advancements in extraction technologies, challenges remain in standardizing and scaling up these processes for industrial applications (More et al., 2022). The reproducibility, efficiency, and sustainability of extraction methods must be addressed to ensure consistency in phytochemical composition and bioactivity across different production batches (Da Silva et al., 2022). Additionally, regulatory frameworks, cost-effectiveness, and environmental considerations play crucial roles in determining the feasibility of large-scale extraction operations (Bouizgma et al., 2025).

### 7.1 Standardization of extraction methods

Achieving standardization in natural product extraction is challenging due to the inherent variability in plant materials, including differences in species, growing conditions, and harvesting times (Bandaranayake, 2006; Afshar et al., 2022). The same extraction method may yield different phytochemical profiles depending on these variables (Wong et al., 2022). Therefore, standardized protocols must be established to ensure batch-to-batch consistency.

Reproducibility Issues: Variability in raw materials affects extraction efficiency, necessitating strict control of processing parameters such as solvent type, temperature, and extraction time (Sridhar et al., 2022).

Optimization of Key Parameters: Developing standardized protocols for solvent concentration, pH, pressure, and extraction duration is critical to achieving reliable results (Risticevic et al., 2010).

Regulatory Compliance: The standardization of extraction methods is essential for meeting pharmaceutical and nutraceutical regulatory guidelines (e.g., FDA, EMA, WHO) (Hossain et al., 2022).

### 7.2 Challenges in scaling up extraction processes

While many extraction techniques are effective in laboratory-scale studies, their industrial-scale implementation presents technical and economic challenges.

Equipment and Infrastructure Limitations: Large-scale extraction requires specialized equipment such as high-pressure supercritical CO_2_ systems or industrial-scale ultrasonic reactors, which involve high capital investment (Duba and Fiori, 2019).

Energy and Solvent Consumption: Scaling up extraction processes can significantly increase energy consumption and solvent use, leading to higher production costs and potential environmental impact (Pai et al., 2022).

Process Efficiency and Yield Optimization: Industrial-scale extractions must be optimized to maximize yield while maintaining bioactivity, ensuring cost-effectiveness without compromising product quality (Yadav et al., 2024).

### 7.3 Environmental and sustainability considerations

The environmental impact of large-scale extraction processes is a growing concern, particularly with the use of organic solvents and excessive energy consumption.

Green Extraction Technologies: Sustainable alternatives such as supercritical CO_2_ extraction, pressurized liquid extraction, and enzymatic-assisted extraction are being explored to minimize solvent waste and energy use (Alexandre et al., 2018).

Solvent-Free and Low-Energy Approaches: Techniques like subcritical water extraction and membrane-based separation systems offer eco-friendly solutions for extracting bioactive compounds with minimal environmental impact (Mondal et al., 2025).

Waste Management and Byproduct Utilization: Developing methods to recycle solvents and utilize extraction byproducts in secondary applications (e.g., animal feed, fertilizers) can enhance the sustainability of extraction operations (Soh and Eckelman, 2016).

### 7.4 Future perspectives and innovations

While significant progress has been made, several key gaps in the literature need to be addressed to advance the field. Future research should be prioritized in the following areas.

• Techno-Economic Analyses (TEA): Direct and rigorous cost-benefit analyses comparing advanced extraction techniques (e.g., SFE vs MAE for a specific high-value compound) are urgently needed (Feng et al., 2022). Such studies are critical for guiding industrial adoption and determining economic viability (Apostolakou et al., 2009).
• Optimization of Hybrid Systems: As integrated extraction strategies show great promise, systematic research is required to optimize the parameters of these multi-step processes (Rodríguez-Pérez et al., 2016). Understanding the synergistic and antagonistic interactions between different techniques is key to maximizing their potential (Macedo et al., 2023).
• Automation and Process Control: While artificial intelligence (AI)-driven optimization shows promise, developing and validating cost-effective, real-time monitoring sensors for industrial-scale extractions remains a key challenge that requires further research (van den Berg et al., 2013; Grassi and Alamprese, 2018).
• Bioavailability Enhancement: Although nanoencapsulation can improve stability, more clinical research is needed to develop scalable, food-grade nano-delivery systems and to confirm their long-term *in vivo* efficacy and safety (McClements, 2014).
• Scalability of Green Solvents: While green solvents like DES are effective at the lab scale, research into their large-scale production, stability, and efficient recycling is still in its early stages and presents a major hurdle for industrial implementation (Socas-Rodríguez et al., 2021).

## 8 Conlusion

The choice of an extraction technique is a critical determinant in harnessing the therapeutic potential of natural products. It governs not only the yield and phytochemical composition of an extract but, more importantly, the preservation of the inherent bioactivity of its constituent compounds. This review has illustrated that while conventional methods are simple, they often compromise compound integrity due to harsh conditions. In contrast, advanced techniques such as UAE, MAE, and SFE offer superior efficiency and preservation, though their effectiveness is highly dependent on rigorous parameter optimization, as underscored by the literature’s variable findings.

Ultimately, this review highlights that the future of natural product valorization lies in the intelligent integration of hybrid strategies to maximize recovery while ensuring sustainability and economic viability. A well-chosen and meticulously optimized extraction strategy is the essential first step in translating the rich chemical diversity of natural products into safe and effective therapeutic agents for pharmaceutical and nutraceutical applications.

## References

[B1] Abbas A. Anwar F. Ahmad N. (2017). Variation in physico-chemical composition and biological attributes of common basil essential oils produced by hydro-distillation and super critical fluid extraction. J. Essent. Oil Bear. Plants 20, 95–109. 10.1080/0972060x.2017.1280418

[B2] Aduloju E. I. Yahaya N. Mohammad Zain N. Anuar Kamaruddin M. Ariffuddin Abd Hamid M. (2023). An overview on the use of deep eutectic solvents for green extraction of some selected bioactive compounds from natural matrices. Adv. J. Chem. Sect. A 6, 253–300.

[B3] Afshar M. Najafian S. Radi M. (2022). The effect of harvest time on the natural product of *Rosmarinus officinalis* L. from South Iran (Fars province). Nat. Prod. Res. 36, 2637–2642. 10.1080/14786419.2021.1914615 33974457

[B4] Ahangari H. King J. W. Ehsani A. Yousefi M. (2021). Supercritical fluid extraction of seed oils–A short review of current trends. Trends Food Sci. Technol. 111, 249–260. 10.1016/j.tifs.2021.02.066

[B5] Ahmad S. Singh A. Akram W. Upadhyay A. Abrol G. S. (2025). Algal lipids: a review on current status and future prospects in food processing. J. Food Sci. 90, e17618. 10.1111/1750-3841.17618 39786345

[B6] Ahmed F. Eticha T. Hymete A. Ashenef A. (2023). “Principles and applications of ultra-high-performance liquid chromatography,” in High performance liquid chromatography-recent advances and applications (IntechOpen).

[B7] Alexandre E. M. Moreira S. A. Castro L. M. Pintado M. Saraiva J. A. (2018). Emerging technologies to extract high added value compounds from fruit residues: sub/supercritical, ultrasound-and enzyme-assisted extractions. Food Rev. Int. 34, 581–612. 10.1080/87559129.2017.1359842

[B8] Al-Khayri J. M. Sahana G. R. Nagella P. Joseph B. V. Alessa F. M. Al-Mssallem M. Q. (2022). Flavonoids as potential anti-inflammatory molecules: a review. Molecules 27, 2901. 10.3390/molecules27092901 35566252 PMC9100260

[B9] Altemimi A. Lakhssassi N. Baharlouei A. Watson D. G. Lightfoot D. A. (2017). Phytochemicals: extraction, isolation, and identification of bioactive compounds from plant extracts. Plants 6, 42. 10.3390/plants6040042 28937585 PMC5750618

[B10] Ameer K. Shahbaz H. M. Kwon J. H. (2017). Green extraction methods for polyphenols from plant matrices and their byproducts: a review. Compr. Rev. Food Sci. food Saf. 16, 295–315. 10.1111/1541-4337.12253 33371540

[B11] Anaya-Esparza L. M. Aurora-Vigo E. F. Villagrán Z. Rodríguez-Lafitte E. Ruvalcaba-Gómez J. M. Solano-Cornejo M. Á. (2023). Design of experiments for optimizing ultrasound-assisted extraction of bioactive compounds from plant-based sources. Molecules 28, 7752. 10.3390/molecules28237752 38067479 PMC10707804

[B12] Antony A. Farid M. (2022). Effect of temperatures on polyphenols during extraction. Appl. Sci. 12, 2107. 10.3390/app12042107

[B13] Apostolakou A. Kookos I. Marazioti C. Angelopoulos K. (2009). Techno-economic analysis of a biodiesel production process from vegetable oils. Fuel Process. Technol. 90, 1023–1031. 10.1016/j.fuproc.2009.04.017

[B14] Atanasov A. G. Zotchev S. B. Dirsch V. M. Supuran C. T. (2021). Natural products in drug discovery: advances and opportunities. Nat. Rev. Drug Discov. 20, 200–216. 10.1038/s41573-020-00114-z 33510482 PMC7841765

[B15] Awad A. M. Kumar P. Ismail-Fitry M. R. Jusoh S. Ab Aziz M. F. Sazili A. Q. (2021). Green extraction of bioactive compounds from plant biomass and their application in meat as natural antioxidant. Antioxidants 10, 1465. 10.3390/antiox10091465 34573097 PMC8466011

[B16] Azwanida N. (2015). A review on the extraction methods use in medicinal plants, principle, strength and limitation. Med. Aromat. plants 4, 2167–0412.

[B17] Baker M. J. Trevisan J. Bassan P. Bhargava R. Butler H. J. Dorling K. M. (2014). Using Fourier transform IR spectroscopy to analyze biological materials. Nat. Protoc. 9, 1771–1791. 10.1038/nprot.2014.110 24992094 PMC4480339

[B18] Bandaranayake W. M. (2006). Quality control, screening, toxicity, and regulation of herbal drugs. Mod. Phytomedicine Turn. Med. Plants Drugs, 25–57. 10.1002/9783527609987.ch2

[B19] Beale D. J. Pinu F. R. Kouremenos K. A. Poojary M. M. Narayana V. K. Boughton B. A. (2018). Review of recent developments in GC–MS approaches to metabolomics-based research. Metabolomics 14, 152–31. 10.1007/s11306-018-1449-2 30830421

[B20] Belščak-Cvitanović A. Komes D. (2017). “Extraction and formulation of bioactive compounds,” in Handbook of coffee processing by-products (Elsevier), 93–140.

[B21] Bezerra M. A. Santelli R. E. Oliveira E. P. Villar L. S. Escaleira L. A. (2008). Response surface methodology (RSM) as a tool for optimization in analytical chemistry. Talanta 76, 965–977. 10.1016/j.talanta.2008.05.019 18761143

[B22] Bhadange Y. A. Carpenter J. Saharan V. K. (2024). A comprehensive review on advanced extraction techniques for retrieving bioactive components from natural sources. ACS omega 9, 31274–31297. 10.1021/acsomega.4c02718 39072073 PMC11270575

[B23] Boligon A. A. Athayde M. L. (2014). Importance of HPLC in analysis of plants extracts. Austin Chromatogr. 1, 2.

[B24] Bosiljkov T. Dujmić F. Bubalo M. C. Hribar J. Vidrih R. Brnčić M. (2017). Natural deep eutectic solvents and ultrasound-assisted extraction: green approaches for extraction of wine lees anthocyanins. Food Bioprod. Process. 102, 195–203. 10.1016/j.fbp.2016.12.005

[B25] Bouizgma K. Rabbah N. Abbas Z. Abourriche A. (2025). Unlocking sustainable extraction of natural antioxidants: green solvents, smart technologies, scalability and future directions. Sep. Sci. Technol. 60, 657–683. 10.1080/01496395.2025.2452411

[B26] Brglez Mojzer E. Knez Hrnčič M. Škerget M. Knez Ž. Bren U. (2016). Polyphenols: extraction methods, antioxidative action, bioavailability and anticarcinogenic effects. Molecules 21, 901. 10.3390/molecules21070901 27409600 PMC6273793

[B27] Butler M. S. (2004). The role of natural product chemistry in drug discovery. J. Nat. Prod. 67, 2141–2153. 10.1021/np040106y 15620274

[B28] Cade-Menun B. J. (2005). Using phosphorus-31 nuclear magnetic resonance spectroscopy to characterize organic phosphorus in environmental samples. Org. Phosphorus Environ., 21–44. 10.1079/9780851998220.0021

[B29] Calderón-Oliver M. Ponce-Alquicira E. (2021). Environmentally friendly techniques and their comparison in the extraction of natural antioxidants from green tea, rosemary, clove, and oregano. Molecules 26, 1869. 10.3390/molecules26071869 33810281 PMC8036828

[B30] Camel V. (2000). Microwave-assisted solvent extraction of environmental samples. TrAC Trends Anal. Chem. 19, 229–248. 10.1016/s0165-9936(99)00185-5

[B31] Chemat F. Abert-Vian M. Fabiano-Tixier A. S. Strube J. Uhlenbrock L. Gunjevic V. (2019a). Green extraction of natural products. Origins, current status, and future challenges. TrAC Trends Anal. Chem. 118, 248–263. 10.1016/j.trac.2019.05.037

[B32] Chemat F. Abert Vian M. Ravi H. K. Khadhraoui B. Hilali S. Perino S. (2019b). Review of alternative solvents for green extraction of food and natural products: Panorama, principles, applications and prospects. Molecules 24, 3007. 10.3390/molecules24163007 31430982 PMC6721174

[B33] Chemat F. Rombaut N. Sicaire A.-G. Meullemiestre A. Fabiano-Tixier A.-S. Abert-Vian M. (2017). Ultrasound assisted extraction of food and natural products. Mechanisms, techniques, combinations, protocols and applications. A review. Ultrason. Sonochemistry 34, 540–560. 10.1016/j.ultsonch.2016.06.035 27773280

[B34] Chemat F. Vian M. A. Fabiano-Tixier A.-S. Nutrizio M. Jambrak A. R. Munekata P. E. (2020). A review of sustainable and intensified techniques for extraction of food and natural products. Green Chem. 22, 2325–2353. 10.1039/c9gc03878g

[B35] Cheng Y. Xue F. Yu S. Du S. Yang Y. (2021). Subcritical water extraction of natural products. Molecules 26, 4004. 10.3390/molecules26134004 34209151 PMC8271798

[B36] Chongo Y. (2025). Extraction methods of bioactive compounds: a sustainability approach. J. Food Sci. Gastron. 3, 29–37.

[B37] Choudhary N. Sekhon B. S. (2011). An overview of advances in the standardization of herbal drugs. J. Pharm. Educ. Res. 2, 55.

[B38] Chuo S. C. Nasir H. M. Mohd-Setapar S. H. Mohamed S. F. Ahmad A. Wani W. A. (2022). A glimpse into the extraction methods of active compounds from plants. Crit. Rev. Anal. Chem. 52, 667–696. 10.1080/10408347.2020.1820851 32954795

[B39] Costa J. R. Tonon R. V. Cabral L. Gottschalk L. Pastrana L. Pintado M. E. (2020). Valorization of agricultural lignocellulosic plant byproducts through enzymatic and enzyme-assisted extraction of high-value-added compounds: a review. ACS Sustain. Chem. Eng. 8, 13112–13125. 10.1021/acssuschemeng.0c02087

[B40] Ćujić N. Šavikin K. Janković T. Pljevljakušić D. Zdunić G. Ibrić S. (2016). Optimization of polyphenols extraction from dried chokeberry using maceration as traditional technique. Food Chem. 194, 135–142. 10.1016/j.foodchem.2015.08.008 26471536

[B41] Da Cunha L. N. L. Tizziani T. Souza G. B. Moreira M. A. Neto J. S. Dos Santos C. V. (2022). Natural products with tandem anti-inflammatory, immunomodulatory and anti-SARS-CoV/2 effects: a drug discovery perspective against SARS-CoV-2. Curr. Med. Chem. 29, 2530–2564. 10.2174/0929867328666210726094955 34313197

[B42] Dai Y. Van Spronsen J. Witkamp G.-J. Verpoorte R. Choi Y. H. (2013). Natural deep eutectic solvents as new potential media for green technology. Anal. Chim. acta 766, 61–68. 10.1016/j.aca.2012.12.019 23427801

[B43] Dashtian K. Kamalabadi M. Ghoorchian A. Ganjali M. R. Rahimi-Nasrabadi M. (2024). Integrated supercritical fluid extraction of essential oils. J. Chromatogr. A 1733, 465240. 10.1016/j.chroma.2024.465240 39154494

[B44] Da Silva B. V. Barreira J. C. Oliveira M. B. P. (2016a). Natural phytochemicals and probiotics as bioactive ingredients for functional foods: extraction, biochemistry and protected-delivery technologies. Trends Food Sci. Technol. 50, 144–158. 10.1016/j.tifs.2015.12.007

[B45] Da Silva R. F. Carneiro C. N. De Sousa C. B. D. C. Gomez F. J. Espino M. Boiteux J. (2022). Sustainable extraction bioactive compounds procedures in medicinal plants based on the principles of green analytical chemistry: a review. Microchem. J. 175, 107184. 10.1016/j.microc.2022.107184

[B46] Da Silva R. P. Rocha-Santos T. A. Duarte A. C. (2016b). Supercritical fluid extraction of bioactive compounds. TrAC Trends Anal. Chem. 76, 40–51. 10.1016/j.trac.2015.11.013

[B47] David Troncoso F. Alberto Sánchez D. Luján Ferreira M. (2022). Production of plant proteases and new biotechnological applications: an updated review. ChemistryOpen 11, e202200017. 10.1002/open.202200017 35286022 PMC8919702

[B48] De Melo M. Silvestre A. Silva C. (2014). Supercritical fluid extraction of vegetable matrices: applications, trends and future perspectives of a convincing green technology. J. Supercrit. Fluids 92, 115–176. 10.1016/j.supflu.2014.04.007

[B49] Demesa A. G. Saavala S. Pöysä M. Koiranen T. (2024). Overview and toxicity assessment of ultrasound-assisted extraction of natural ingredients from plants. Foods 13, 3066. 10.3390/foods13193066 39410101 PMC11476364

[B50] De Souza T. S. Kawaguti H. Y. (2021). Cellulases, hemicellulases, and pectinases: applications in the food and beverage industry. Food Bioprocess Technol. 14, 1446–1477. 10.1007/s11947-021-02678-z

[B51] Dhobi M. Mandal V. Hemalatha S. (2009). Optimization of microwave assisted extraction of bioactive flavonolignan-silybinin. J. Chem. Metrol. 3, 13.

[B52] Dhotre I. (2025). A comprehensive review on progression and innovations in microwave assisted extraction technology for essential oils. J. Chem. Technol. Biotechnol. 100, 894–907. 10.1002/jctb.7823

[B53] Dong M. (2013). The essence of modern HPLC: advantages, limitations, fundamentals, and opportunities.

[B54] Dong M. W. Zhang K. (2014). Ultra-high-pressure liquid chromatography (UHPLC) in method development. TrAC Trends Anal. Chem. 63, 21–30. 10.1016/j.trac.2014.06.019

[B55] Doughari J. H. (2012). Phytochemicals: extraction methods, basic structures and mode of action as potential chemotherapeutic agents. Rijeka, Croatia: INTECH Open Access Publisher.

[B56] Duba K. Fiori L. (2019). Supercritical CO_2_ extraction of grape seeds oil: scale‐up and economic analysis. Int. J. Food Sci. Technol. 54, 1306–1312. 10.1111/ijfs.14104

[B57] Durazzo A. Sorkin B. C. Lucarini M. Gusev P. A. Kuszak A. J. Crawford C. (2022). Analytical challenges and metrological approaches to ensuring dietary supplement quality: international perspectives. Front. Pharmacol. 12, 714434. 10.3389/fphar.2021.714434 35087401 PMC8787362

[B58] Enaru B. Drețcanu G. Pop T. D. Stǎnilǎ A. Diaconeasa Z. (2021). Anthocyanins: factors affecting their stability and degradation. Antioxidants 10, 1967. 10.3390/antiox10121967 34943070 PMC8750456

[B59] Feng L. Liu J. Lu H. Liu B. Chen Y. (2022). Techno-economic and profitability analysis of plant for producing biodiesel from fresh vegetable oil and waste frying oil on large-scale. Fuel 323, 124304. 10.1016/j.fuel.2022.124304

[B60] Ferreira C. Sarraguça M. (2024). A comprehensive review on deep eutectic solvents and its use to extract bioactive compounds of pharmaceutical interest. Pharmaceuticals 17, 124. 10.3390/ph17010124 38256957 PMC10820243

[B61] Foroutani Z. Mogaddam M. R. A. Ghasempour Z. Ghareaghajlou N. (2024). Application of deep eutectic solvents in the extraction of anthocyanins: stability, bioavailability, and antioxidant property. Trends Food Sci. Technol. 144, 104324. 10.1016/j.tifs.2023.104324

[B62] Friedman M. Jürgens H. S. (2000). Effect of pH on the stability of plant phenolic compounds. J. Agric. Food Chem. 48, 2101–2110. 10.1021/jf990489j 10888506

[B63] Gallego R. Bueno M. Herrero M. (2019). Sub-and supercritical fluid extraction of bioactive compounds from plants, food-by-products, seaweeds and microalgae–An update. TrAC Trends Anal. Chem. 116, 198–213. 10.1016/j.trac.2019.04.030

[B64] Gautam V. Garg V. Meena N. Kumari S. Patel S. Singh H. (2025). Harnessing NMR technology for enhancing field crop improvement: applications, challenges, and future perspectives. Metabolomics 21, 27. 10.1007/s11306-025-02229-z 39979661

[B65] Golmakani M.-T. Rezaei K. (2008). Comparison of microwave-assisted hydrodistillation withthe traditional hydrodistillation method in the extractionof essential oils from *Thymus vulgaris* L. Food Chem. 109, 925–930. 10.1016/j.foodchem.2007.12.084 26050009

[B66] Grassi S. Alamprese C. (2018). Advances in NIR spectroscopy applied to process analytical technology in food industries. Curr. Opin. Food Sci. 22, 17–21. 10.1016/j.cofs.2017.12.008

[B67] Gurib-Fakim A. (2006). Medicinal plants: traditions of yesterday and drugs of tomorrow. Mol. Aspects Med. 27, 1–93. 10.1016/j.mam.2005.07.008 16105678

[B68] Hao C. Zhao X. Yang P. (2007). GC-MS and HPLC-MS analysis of bioactive pharmaceuticals and personal-care products in environmental matrices. TrAC Trends Anal. Chem. 26, 569–580. 10.1016/j.trac.2007.02.011

[B69] Herrero M. Mendiola J. A. Cifuentes A. Ibáñez E. (2010). Supercritical fluid extraction: recent advances and applications. J. Chromatogr. A 1217, 2495–2511. 10.1016/j.chroma.2009.12.019 20022016

[B70] Herzyk F. Piłakowska-Pietras D. Korzeniowska M. (2024). Supercritical extraction techniques for obtaining biologically active substances from a variety of plant byproducts. Foods 13, 1713. 10.3390/foods13111713 38890941 PMC11171758

[B71] Hessel V. Tran N. N. Asrami M. R. Tran Q. D. Long N. V. D. Escribà-Gelonch M. (2022). Sustainability of green solvents–review and perspective. Green Chem. 24, 410–437. 10.1039/d1gc03662a

[B72] Hidayat R. Wulandari P. (2021). Methods of extraction: maceration, percolation and decoction. Eureka Herba Indones. 2, 68–74.

[B73] Hossain C. M. Gera M. Ali K. A. (2022). Current status and challenges of herbal drug development and regulatory aspect: a global perspective. Asian J. Pharm. Clin. Res. 15, 31–41. 10.22159/ajpcr.2022.v15i12.46134

[B75] Hubschmann H.-J. (2025). Handbook of GC-MS: fundamentals and applications. John Wiley and Sons.

[B76] Huck C. (2015). “Infrared spectroscopic technologies for the quality control of herbal medicines,” in Evidence-based validation of herbal medicine (Elsevier), 477–493.

[B77] Ingle K. P. Deshmukh A. G. Padole D. A. Dudhare M. S. Moharil M. P. Khelurkar V. C. (2017). Phytochemicals: extraction methods, identification and detection of bioactive compounds from plant extracts. J. Pharmacogn. Phytochemistry 6, 32–36.

[B78] Islam M. Malakar S. Rao M. V. Kumar N. Sahu J. K. (2023). Recent advancement in ultrasound-assisted novel technologies for the extraction of bioactive compounds from herbal plants: a review. Food Sci. Biotechnol. 32, 1763–1782. 10.1007/s10068-023-01346-6 37781053 PMC10541372

[B79] Jeong K. M. Zhao J. Jin Y. Heo S. R. Han S. Y. Yoo D. E. (2015). Highly efficient extraction of anthocyanins from grape skin using deep eutectic solvents as green and tunable media. Archives Pharmacal Res. 38, 2143–2152. 10.1007/s12272-015-0678-4 26534763

[B80] Kanitkar A. Sabliov C. Balasubramanian S. Lima M. Boldor D. (2011). Microwave-assisted extraction of soybean and rice bran oil: yield and extraction kinetics. Trans. ASABE 54, 1387–1394. 10.13031/2013.39007

[B81] Khan M. K. Abert-Vian M. Fabiano-Tixier A.-S. Dangles O. Chemat F. (2010). Ultrasound-assisted extraction of polyphenols (flavanone glycosides) from orange (*Citrus sinensis* L.) peel. Food Chem. 119, 851–858. 10.1016/j.foodchem.2009.08.046

[B82] Khaw K.-Y. Parat M.-O. Shaw P. N. Falconer J. R. (2017). Solvent supercritical fluid technologies to extract bioactive compounds from natural sources: a review. Molecules 22, 1186. 10.3390/molecules22071186 28708073 PMC6152233

[B83] Kornilova T. A. Ukolov A. I. Kostikov R. R. Zenkevich I. G. (2013). A simple criterion for gas chromatography/mass spectrometric analysis of thermally unstable compounds, and reassessment of the by‐products of alkyl diazoacetate synthesis. Rapid Commun. Mass Spectrom. 27, 461–466. 10.1002/rcm.6457 23280978

[B84] Kratky L. Jirout T. (2011). Biomass size reduction machines for enhancing biogas production. Chem. Eng. Technol. 34, 391–399. 10.1002/ceat.201000357

[B85] Kumar A. P N. Kumar M. Jose A. Tomer V. Oz E. (2023). Major phytochemicals: recent advances in health benefits and extraction method. Molecules 28, 887. 10.3390/molecules28020887 36677944 PMC9862941

[B86] Kumar K. Srivastav S. Sharanagat V. S. (2021). Ultrasound assisted extraction (UAE) of bioactive compounds from fruit and vegetable processing by-products: a review. Ultrason. Sonochemistry 70, 105325. 10.1016/j.ultsonch.2020.105325 PMC7786612 32920300

[B87] Kumari A. Kaur J. Bhattacharyya S. (2018). Application of fourier transform-infrared spectroscopy as a tool for early cancer detection. Am. J. Biomed. Sci. 10, 139–148. 10.5099/aj180300139

[B88] Kunle O. F. Egharevba H. O. Ahmadu P. O. (2012). Standardization of herbal medicines-A review. Int. J. Biodivers. Conservation 4, 101–112. 10.5897/ijbc11.163

[B89] Kuosmanen K. Lehmusjärvi M. Hyötyläinen T. Jussila M. Riekkola M. L. (2003). Factors affecting microporous membrane liquid‐liquid extraction. J. Sep. Sci. 26, 893–902. 10.1002/jssc.200301481

[B90] Lainez-Cerón E. Jiménez-Munguía M. T. López-Malo A. Ramírez-Corona N. (2021). Effect of process variables on heating profiles and extraction mechanisms during hydrodistillation of eucalyptus essential oil. Heliyon 7, e08234. 10.1016/j.heliyon.2021.e08234 34754975 PMC8564561

[B91] Letertre M. P. Dervilly G. Giraudeau P. (2020). Combined nuclear magnetic resonance spectroscopy and mass spectrometry approaches for metabolomics. Anal. Chem. 93, 500–518. 10.1021/acs.analchem.0c04371 33155816

[B92] López-Bascón M. De Castro M. L. (2020). “Soxhlet extraction,” in Liquid-phase extraction (Elsevier), 327–354.

[B93] Lovrić V. Putnik P. Bursać Kovačević D. Jukić M. Dragović-Uzelac V. (2017). Effect of microwave-assisted extraction on the phenolic compounds and antioxidant capacity of blackthorn flowers. Food Technol. Biotechnol. 55, 243–250. 10.17113/ftb.55.02.17.4687 28867955 PMC5569351

[B94] Lucchesi M. E. Chemat F. Smadja J. (2004). Solvent-free microwave extraction of essential oil from aromatic herbs: comparison with conventional hydro-distillation. J. Chromatogr. 1043, 323–327. 10.1016/j.chroma.2004.05.083 15330107

[B95] Luksta I. Spalvins K. (2023). Methods for extraction of bioactive compounds from products: a review. Rigas Teh. Univ. Zinat. Raksti 27, 422–437. 10.2478/rtuect-2023-0031

[B96] Macedo G. A. Barbosa P. D. P. Dias F. F. Crawford L. M. Wang S. C. Bell J. M. D. M. (2023). Optimizing the integration of microwave processing and enzymatic extraction to produce polyphenol-rich extracts from olive pomace. Foods 12, 3754. 10.3390/foods12203754 37893645 PMC10606511

[B97] Malabadi R. B. Kolkar K. P. Chalannavar R. K. Baijnath H. (2024). Cannabis sativa: extraction methods for Phytocannabinoids-An update. World J. Biol. Pharm. Health Sci. 20, 018–058. 10.30574/wjbphs.2024.20.3.0962

[B98] Mandal V. Mohan Y. Hemalatha S. (2007). Microwave assisted extraction—an innovative and promising extraction tool for medicinal plant research. Pharmacogn. Rev. 1, 7–18.

[B99] Manousi N. Sarakatsianos I. Samanidou V. (2019). “Extraction techniques of phenolic compounds and other bioactive compounds from medicinal and aromatic plants,” in Engineering tools in the beverage industry (Elsevier), 283–314.

[B100] Marcone M. F. Wang S. Albabish W. Nie S. Somnarain D. Hill A. (2013). Diverse food-based applications of nuclear magnetic resonance (NMR) technology. Food Res. Int. 51, 729–747. 10.1016/j.foodres.2012.12.046

[B101] Maslov O. Kolisnyk S. Komisarenko M. Golik M. (2022). Study of total antioxidant activity of green tea leaves (*Camellia sinensis* L.). Herba Pol. 68, 1–9. 10.2478/hepo-2022-0003

[B102] Mcclements D. J. (2014). Nanoparticle-and microparticle-based delivery systems: encapsulation, protection and release of active compounds. CRC Press.

[B103] Mondal A. Singh R. K. Sinhamahapatra A. (2025). “Green and sustainable separation processes for environmental and chemical engineering,” in Advances in separation sciences (Elsevier), 457–479.

[B104] More P. R. Jambrak A. R. Arya S. S. (2022). Green, environment-friendly and sustainable techniques for extraction of food bioactive compounds and waste valorization. Trends Food Sci. Technol. 128, 296–315. 10.1016/j.tifs.2022.08.016

[B105] Muniglia L. Claisse N. Baudelet P.-H. Ricochon G. (2014). “Enzymatic aqueous extraction (EAE),” in Alternative solvents for natural products extraction. Berlin, Heidelberg, Germany: Springer-Verlag, 167–204.

[B106] Natolino A. Passaghe P. Brugnera G. Comuzzo P. (2024). Intensification of grape marc (Vitis vinifera) exploitation by subcritical water-ethanol extraction: effect on polyphenol bioactivities and kinetic modelling. J. Food Eng. 381, 112185. 10.1016/j.jfoodeng.2024.112185

[B107] Nde D. B. Foncha A. C. (2020). Optimization methods for the extraction of vegetable oils: a review. Processes 8, 209. 10.3390/pr8020209

[B108] Nikam P. H. Kareparamban J. Jadhav A. Kadam V. (2012). Future trends in standardization of herbal drugs. J. Appl. Pharm. Sci., 38–44.

[B109] Oreopoulou A. Tsimogiannis D. Oreopoulou V. (2019). Extraction of polyphenols from aromatic and medicinal plants: an overview of the methods and the effect of extraction parameters. Polyphenols Plants 243–259. 10.1016/b978-0-12-813768-0.00025-6

[B110] Othman N. A. Idris S. A. Rosli N. R. (2024). “Maceration and Soxhlet extraction of Orthosiphon stamineus–A comparative study,” in AIP conference proceedings (AIP Publishing).

[B111] Oulahal N. Degraeve P. (2022). Phenolic-rich plant extracts with antimicrobial activity: an alternative to food preservatives and biocides? Front. Microbiol. 12, 753518. 10.3389/fmicb.2021.753518 35058892 PMC8764166

[B112] Pai S. Hebbar A. Selvaraj S. (2022). A critical look at challenges and future scopes of bioactive compounds and their incorporations in the food, energy, and pharmaceutical sector. Environ. Sci. Pollut. Res. 29, 35518–35541. 10.1007/s11356-022-19423-4 PMC9079019 35233673

[B113] Paini J. Midolo G. Valenti F. Ottolina G. (2025). One-Pot combined hydrodistillation of industrial orange peel waste for essential oils and pectin recovery: a multi-objective optimization Study. Sustainability 17, 293. 10.3390/su17010293

[B114] Parhiz H. Roohbakhsh A. Soltani F. Rezaee R. Iranshahi M. (2015). Antioxidant and anti‐inflammatory properties of the citrus flavonoids hesperidin and hesperetin: an updated review of their molecular mechanisms and experimental models. Phytotherapy Res. 29, 323–331. 10.1002/ptr.5256 25394264

[B115] Passos F. Carretero J. Ferrer I. (2015). Comparing pretreatment methods for improving microalgae anaerobic digestion: thermal, hydrothermal, microwave and ultrasound. Chem. Eng. J. 279, 667–672. 10.1016/j.cej.2015.05.065

[B116] Patra J. K. Das G. Lee S. Kang S.-S. Shin H.-S. (2018). Selected commercial plants: a review of extraction and isolation of bioactive compounds and their pharmacological market value. Trends Food Sci. Technol. 82, 89–109. 10.1016/j.tifs.2018.10.001

[B117] Paucar L. O. C. Veggi P. C. Viganó J. Meireles M. a.A. (2023). “Supercritical fluid extraction,” in Green extraction techniques in food analysis (Bentham Science Publishers), 280–323.

[B118] Perović A. B. Karabegović I. T. Krstić M. S. Veličković A. V. Avramović J. M. Danilović B. R. (2024). Novel hydrodistillation and steam distillation methods of essential oil recovery from lavender: a comprehensive review. Industrial Crops Prod. 211, 118244. 10.1016/j.indcrop.2024.118244

[B119] Pheko-Ofitlhile T. Makhzoum A. (2024). Impact of hydrodistillation and steam distillation on the yield and chemical composition of essential oils and their comparison with modern isolation techniques. J. Essent. Oil Res. 36, 105–115. 10.1080/10412905.2024.2320350

[B120] Plaskova A. Mlcek J. (2023). New insights of the application of water or ethanol-water plant extract rich in active compounds in food. Front. Nutr. 10, 1118761. 10.3389/fnut.2023.1118761 37057062 PMC10086256

[B121] Prabhune A. Dey R. (2023). Green and sustainable solvents of the future: deep eutectic solvents. J. Mol. Liq. 379, 121676. 10.1016/j.molliq.2023.121676

[B122] Puri M. Sharma D. Barrow C. J. (2012). Enzyme-assisted extraction of bioactives from plants. Trends Biotechnol. 30, 37–44. 10.1016/j.tibtech.2011.06.014 21816495

[B123] Rajha H. N. El Darra N. Hobaika Z. Boussetta N. Vorobiev E. Maroun R. G. (2014). Extraction of total phenolic compounds, flavonoids, anthocyanins and tannins from grape byproducts by response surface methodology. Influence of solid-liquid ratio, particle size, time, temperature and solvent mixtures on the optimization process. Food Nutr. Sci. 5, 333–345.

[B124] Rasul M. G. (2018). Conventional extraction methods use in medicinal plants, their advantages and disadvantages. Int. J. Basic Sci. Appl. Comput. 2, 10–14.

[B125] Rebiai A. Hemmami H. Zeghoud S. Ben Seghir B. Kouadri I. Eddine L. S. (2022). Current application of chemometrics analysis in authentication of natural products: a review. Comb. Chem. High Throughput Screen. 25, 945–972. 10.2174/1386207324666210309102239 33687892

[B126] Reis D. Silva P. Perestrelo R. Câmara J. S. (2020). Residue analysis of insecticides in potatoes by QuEChERS-dSPE/UHPLC-PDA. Foods 9, 1000. 10.3390/foods9081000 32722562 PMC7466252

[B127] Rezvankhah A. Emam-Djomeh Z. Askari G. (2020). Encapsulation and delivery of bioactive compounds using spray and freeze-drying techniques: a review. Dry. Technol. 38, 235–258. 10.1080/07373937.2019.1653906

[B128] Rifna E. Misra N. Dwivedi M. (2023). Recent advances in extraction technologies for recovery of bioactive compounds derived from fruit and vegetable waste peels: a review. Crit. Rev. Food Sci. Nutr. 63, 719–752. 10.1080/10408398.2021.1952923 34309440

[B129] Risticevic S. Lord H. Gorecki T. Arthur C. L. Pawliszyn J. (2010). Protocol for solid-phase microextraction method development. Nat. Protoc. 5, 122–139. 10.1038/nprot.2009.179 20057384

[B130] Rocchetti G. Gregorio R. P. Lorenzo J. M. Barba F. J. Oliveira P. G. Prieto M. A. (2022). Functional implications of bound phenolic compounds and phenolics–food interaction: a review. Compr. Rev. Food Sci. Food Saf. 21, 811–842. 10.1111/1541-4337.12921 35150191

[B131] Rodríguez‐Pérez C. Gilbert‐López B. Mendiola J. A. Quirantes‐Piné R. Segura‐Carretero A. Ibáñez E. (2016). Optimization of microwave‐assisted extraction and pressurized liquid extraction of phenolic compounds from Moringa oleifera leaves by multiresponse surface methodology. Electrophoresis 37, 1938–1946. 10.1002/elps.201600071 27122439

[B132] Şahin S. Bilgin M. Dramur M. U. (2011). Investigation of oleuropein content in olive leaf extract obtained by supercritical fluid extraction and soxhlet methods. Sep. Sci. Technol. 46, 1829–1837. 10.1080/01496395.2011.573519

[B133] Sethi S. Rathod V. (2024). Recent advancements in ultrasound-assisted biomolecule extraction from prokaryotic and eukaryotic cells: a review. Prep. Biochem. Biotechnol., 1–27. 10.1080/10826068.2024.2436952 39718248

[B134] Shanmugam K. R. Shanmugam B. Subbaiah G. V. Ravi S. Reddy K. S. (2021). Medicinal plants and bioactive compounds for diabetes management: important advances in drug discovery. Curr. Pharm. Des. 27, 763–774. 10.2174/1381612826666200928160357 32988345

[B135] Shikov A. N. Mikhailovskaya I. Y. Narkevich I. A. Flisyuk E. V. Pozharitskaya O. N. (2022). “Methods of extraction of medicinal plants,” in Evidence-Based validation of herbal medicine (Elsevier), 771–796.

[B136] Shirsath S. Sonawane S. Gogate P. (2012). Intensification of extraction of natural products using ultrasonic irradiations—A review of current status. Chem. Eng. Process. Process Intensif. 53, 10–23. 10.1016/j.cep.2012.01.003

[B137] Sicker D. Zeller K.-P. Siehl H.-U. Berger S. (2019). Natural products: isolation, structure elucidation, history. John Wiley and Sons.

[B138] Siddiqui T. Sharma V. Khan M. U. Gupta K. (2024). Terpenoids in essential oils: chemistry, classification, and potential impact on human health and industry. Phytomedicine plus 4, 100549. 10.1016/j.phyplu.2024.100549

[B139] Singh M. D. (2017). Nano-fertilizers is a new way to increase nutrients use efficiency in crop production. Int. J. Agric. Sci. 9, 0975–3710.

[B140] Sl Mendez A. Virginia Garcia C. Eb Da Silva F. Reisdorfer Paula F. (2015). Identification and quantification methodologies for active substances in natural products: the hole of chromatographic and spectroscopic techniques. Curr. Chromatogr. 2, 2–19. 10.2174/2213240601666141126205140

[B141] Socas-Rodríguez B. Torres-Cornejo M. V. Álvarez-Rivera G. Mendiola J. A. (2021). Deep eutectic solvents for the extraction of bioactive compounds from natural sources and agricultural by-products. Appl. Sci. 11, 4897. 10.3390/app11114897

[B142] Soh L. Eckelman M. J. (2016). Green solvents in biomass processing. ACS Sustain. Chem. Eng. 4, 5821–5837. 10.1021/acssuschemeng.6b01635

[B143] Song H. Liu J. (2018). GC-O-MS technique and its applications in food flavor analysis. Food Res. Int. 114, 187–198. 10.1016/j.foodres.2018.07.037 30361015

[B144] Sousa A. S. Araújo-Rodrigues H. Pintado M. E. (2023). The health-promoting potential of edible mushroom proteins. Curr. Pharm. Des. 29, 804–823. 10.2174/1381612829666221223103756 36567303

[B145] Spietelun A. Kloskowski A. Chrzanowski W. NamieśNik J. (2013). Understanding solid-phase microextraction: key factors influencing the extraction process and trends in improving the technique. Chem. Rev. 113, 1667–1685. 10.1021/cr300148j 23273266

[B146] Sridhar A. Vaishampayan V. Kumar P. S. Ponnuchamy M. Kapoor A. (2022). Extraction techniques in food industry: insights into process parameters and their optimization. Food Chem. Toxicol. 166, 113207. 10.1016/j.fct.2022.113207 35688271

[B147] Stanisz M. Stanisz B. J. Cielecka-Piontek J. (2024). A comprehensive review on deep eutectic solvents: their current status and potential for extracting active compounds from adaptogenic plants. Molecules 29, 4767. 10.3390/molecules29194767 39407698 PMC11478271

[B148] Suthar K. J. (2025). Natural deep eutectic solvents in extraction science: progress, challenges, and future prospects. Sep. Sci. Plus 8, e70075. 10.1002/sscp.70075

[B149] Tauro S. Dhokchawle B. Mohite P. Nahar D. Nadar S. Coutinho E. (2024). Natural Anticancer agents: their therapeutic potential, challenges and promising outcomes. Curr. Med. Chem. 31, 848–870. 10.2174/0929867330666230502113150 37138435

[B150] Tiwari U. Cummins E. (2013). Factors influencing levels of phytochemicals in selected fruit and vegetables during pre-and post-harvest food processing operations. Food Res. Int. 50, 497–506. 10.1016/j.foodres.2011.09.007

[B151] Tomlinson T. R. Akerele O. (2015). Medicinal plants: their role in health and biodiversity. University of Pennsylvania Press.

[B152] Trolles-Cavalcante S. Y. Dutta A. Sofer Z. Borenstein A. (2021). The effectiveness of Soxhlet extraction as a simple method for GO rinsing as a precursor of high-quality graphene. Nanoscale Adv. 3, 5292–5300. 10.1039/d1na00382h 36132643 PMC9418454

[B153] Tzanova M. Atanasov V. Yaneva Z. Ivanova D. Dinev T. (2020). Selectivity of current extraction techniques for flavonoids from plant materials. Processes 8, 1222. 10.3390/pr8101222

[B154] Usman I. Hussain M. Imran A. Afzaal M. Saeed F. Javed M. (2022). Traditional and innovative approaches for the extraction of bioactive compounds. Int. J. Food Prop. 25, 1215–1233. 10.1080/10942912.2022.2074030

[B155] Usman M. Nakagawa M. Cheng S. (2023). Emerging trends in green extraction techniques for bioactive natural products. Processes 11, 3444. 10.3390/pr11123444

[B156] Valisakkagari H. Chaturvedi C. Rupasinghe H. V. (2024). Green extraction of phytochemicals from fresh vegetable waste and their potential application as cosmeceuticals for skin health. Processes 12, 742. 10.3390/pr12040742

[B157] Van Den Berg F. Lyndgaard C. B. Sørensen K. M. Engelsen S. B. (2013). Process analytical technology in the food industry. Trends Food Sci. Technol. 31, 27–35. 10.1016/j.tifs.2012.04.007

[B158] Ventura S. P. E Silva F. A. Quental M. V. Mondal D. Freire M. G. Coutinho J. A. (2017). Ionic-liquid-mediated extraction and separation processes for bioactive compounds: past, present, and future trends. Chem. Rev. 117, 6984–7052. 10.1021/acs.chemrev.6b00550 28151648 PMC5447362

[B159] Vijayalakshmi R. Ravindhran R. (2012). Comparative fingerprint and extraction yield of *Diospyrus ferrea* (willd.) Bakh. root with phenol compounds (gallic acid), as determined by uv–vis and ft–ir spectroscopy. Asian Pac. J. Trop. Biomed. 2, S1367–S1371. 10.1016/s2221-1691(12)60418-3

[B160] Vinatoru M. Mason T. Calinescu I. (2017). Ultrasonically assisted extraction (UAE) and microwave assisted extraction (MAE) of functional compounds from plant materials. TrAC Trends Anal. Chem. 97, 159–178. 10.1016/j.trac.2017.09.002

[B161] Wang T. Zhu L. Mei L. Kanda H. (2024). Extraction and separation of natural products from microalgae and other natural sources using liquefied dimethyl ether, a green solvent: a review. Foods 13, 352. 10.3390/foods13020352 38275719 PMC10815339

[B162] Wang W. Rao L. Wu X. Wang Y. Zhao L. Liao X. (2021). Supercritical carbon dioxide applications in food processing. Food Eng. Rev. 13, 570–591. 10.1007/s12393-020-09270-9

[B163] Wen L. Zhang Z. Sun D.-W. Sivagnanam S. P. Tiwari B. K. (2020). Combination of emerging technologies for the extraction of bioactive compounds. Crit. Rev. Food Sci. Nutr. 60, 1826–1841. 10.1080/10408398.2019.1602823 30990060

[B164] Wijesinghe W. Jeon Y.-J. (2012). Enzyme-assistant extraction (EAE) of bioactive components: a useful approach for recovery of industrially important metabolites from seaweeds: a review. Fitoterapia 83, 6–12. 10.1016/j.fitote.2011.10.016 22061659

[B165] Wijngaard H. Hossain M. B. Rai D. K. Brunton N. (2012). Techniques to extract bioactive compounds from food by-products of plant origin. Food Res. Int. 46, 505–513. 10.1016/j.foodres.2011.09.027

[B166] Wolfender J.-L. (2009). HPLC in natural product analysis: the detection issue. Planta Medica 75, 719–734. 10.1055/s-0028-1088393 19145552

[B167] Wong M. Sirisena S. Ng K. (2022). Phytochemical profile of differently processed tea: a review. J. Food Sci. 87, 1925–1942. 10.1111/1750-3841.16137 35368105

[B168] Wu H. Guo J. Chen S. Liu X. Zhou Y. Zhang X. (2013). Recent developments in qualitative and quantitative analysis of phytochemical constituents and their metabolites using liquid chromatography–mass spectrometry. J. Pharm. Biomed. Analysis 72, 267–291. 10.1016/j.jpba.2012.09.004 23031576

[B169] Xie C. Yu J. Huang S. Gao W. Tang K. (2019). A novel approach of matching mass-to-charge ratio for compound identification in gas chromatography–mass spectrometry. J. AOAC Int. 102, 638–645. 10.5740/jaoacint.18-0261 30446021

[B74] Xiong L. Hu W.-B. Yang Z.-W. Wang H.-C. Wang N. Liu X. (2019). Enzymolysis-ultrasonic assisted extraction of flavanoid from *Cyclocarya paliurus* (Batal) Iljinskaja: HPLC profile, antimicrobial and antioxidant activity. Industrial Crops Prod. 130, 615–626. 10.1016/j.indcrop.2019.01.027

[B170] Yadav S. Malik K. Moore J. M. Kamboj B. R. Malik S. Malik V. K. (2024). Valorisation of agri-food waste for bioactive compounds: recent trends and future sustainable challenges. Molecules 29, 2055. 10.3390/molecules29092055 38731546 PMC11085133

[B171] Ye J. (2009). Application of gas chromatography-mass spectrometry in research of traditional Chinese medicine. Chem. Pap. 63, 506–511. 10.2478/s11696-009-0056-0

[B172] Yingngam B. Navabhatra A. Sillapapibool P. (2024). “AI-Driven decision-making applications in pharmaceutical sciences,” in Using traditional design methods to enhance AI-Driven decision making. IGI Global Scientific Publishing, 1–63.

[B173] Yolci Omeroglu P. Acoglu B. Özdal T. Tamer C. E. Çopur Ö. U. (2019). “Extraction techniques for plant-based bio-active compounds,” Cham, Switzerland: Springer in Natural bio-active compounds: volume 2: chemistry, pharmacology and health care practices, 465–492.

[B174] Yuan B. Byrnes D. R. Dinssa F. F. Simon J. E. Wu Q. (2019). Identification of polyphenols, glycoalkaloids, and saponins in Solanum scabrum berries using HPLC‐UV/Vis‐MS. J. Food Sci. 84, 235–243. 10.1111/1750-3841.14424 30693503

[B175] Yubin J. Miao Y. Bing W. Yao Z. (2014). The extraction, separation and purification of alkaloids in the natural medicine. J. Chem. Pharm. Res. 6, 338–345.

[B176] Zhang J. Wen C. Zhang H. Duan Y. Ma H. (2020). Recent advances in the extraction of bioactive compounds with subcritical water: a review. Trends Food Sci. Technol. 95, 183–195. 10.1016/j.tifs.2019.11.018

[B177] Zia S. Khan M. R. Shabbir M. A. Aslam Maan A. Khan M. K. I. Nadeem M. (2022). An inclusive overview of advanced thermal and nonthermal extraction techniques for bioactive compounds in food and food-related matrices. Food Rev. Int. 38, 1166–1196. 10.1080/87559129.2020.1772283

[B178] Žuvela P. Skoczylas M. Jay Liu J. Ba̧Czek T. Kaliszan R. Wong M. W. (2019). Column characterization and selection systems in reversed-phase high-performance liquid chromatography. Chem. Rev. 119, 3674–3729. 10.1021/acs.chemrev.8b00246 30604951

